# Clinical Significance of Pain in Differential Diagnosis between Spinal Meningioma and Schwannoma

**DOI:** 10.1155/2020/7947242

**Published:** 2020-06-26

**Authors:** Dorota Olex-Zarychta

**Affiliations:** Institute of Sport Sciences, Academy of Physical Education, Mikolowska 72a 40-065 Katowice, Poland

## Abstract

While common MRI characteristics for schwannomas exist, diagnosis by imaging alone remains challenging. Detailed analysis of symptoms reported by the patient is very important in the differential diagnosis between meningioma and schwannoma in cases where MRI images are not sufficient for determining the tumor type. The pain, its classification of the character, and the timing seem to be an important predictive symptom in the differential diagnosis of spinal schwannoma.

## 1. Introduction

The most common extramedullary intradural spinal tumors are schwannomas (29%) followed by meningiomas (25%) [1]. It is difficult to distinguish these lesions by clinical examination because symptoms associated with spinal tumors are nonspecific. Most commonly schwannomas are seen in cervical and lumbar regions, less frequently in the thoracic spinal segment. Meningiomas commonly occur in the thoracic region (80%). Clinical symptoms from these tumors are usually related to their size and anatomic location. The most common initial symptom is pain, followed by a loss of nerve function [2]. The pain can be characterized as back pain, which may be local, nocturnal, or radiating to the arms or legs [3]. Pain occurs in 52-80% of all patients, with a higher incidence in schwannomas than in meningiomas. Smaller tumors, especially meningiomas, may be painless for a patient. Among neurological symptoms of spinal tumors, progressive weakness, numbness in the legs, and urinary incontinence are common. Sphincter dysfunction, paraparesis, and erectile dysfunction occur in 20%, 12%, and 2% of patients, respectively [3].

The primary diagnostic modality for intradural tumors is magnetic resonance imaging (MRI) without and with contrast enhancement. However, differentiation between meningioma and schwannoma on the basis of MR image may be difficult without additional data from detailed anamnesis of the patient and symptom analysis. The typical magnetic resonance (MR) appearance of schwannoma is iso-to-hyperintense on T_1_-weighted images, hyperintense on fluid-sensitive sequences, and often diffusely enhancing on contrast-enhanced images [4]. Spinal meningiomas are iso- or hypointense on T_1_-weighted images and slightly hyperintense or hypointense on T_2_-weighted MRI. Upon contrast application, they enhance vividly (except for a calcified part) and frequently display a “dural tail” sign. About 5% of meningiomas may present in a dumbbell shape [3].

The aim of this paper is to present a diagnostically challenging case of intradural spinal tumor and to provide some useful guidelines for differential diagnosis between spinal schwannoma and meningioma related to preoperative pain characteristics. Pain is considered as a classic symptom of spinal tumors; however, there has been little discussion regarding the clinical significance of pain in differential diagnosis and its relevance in preoperative planning.

## 2. Case Presentation

**Figure 1:**
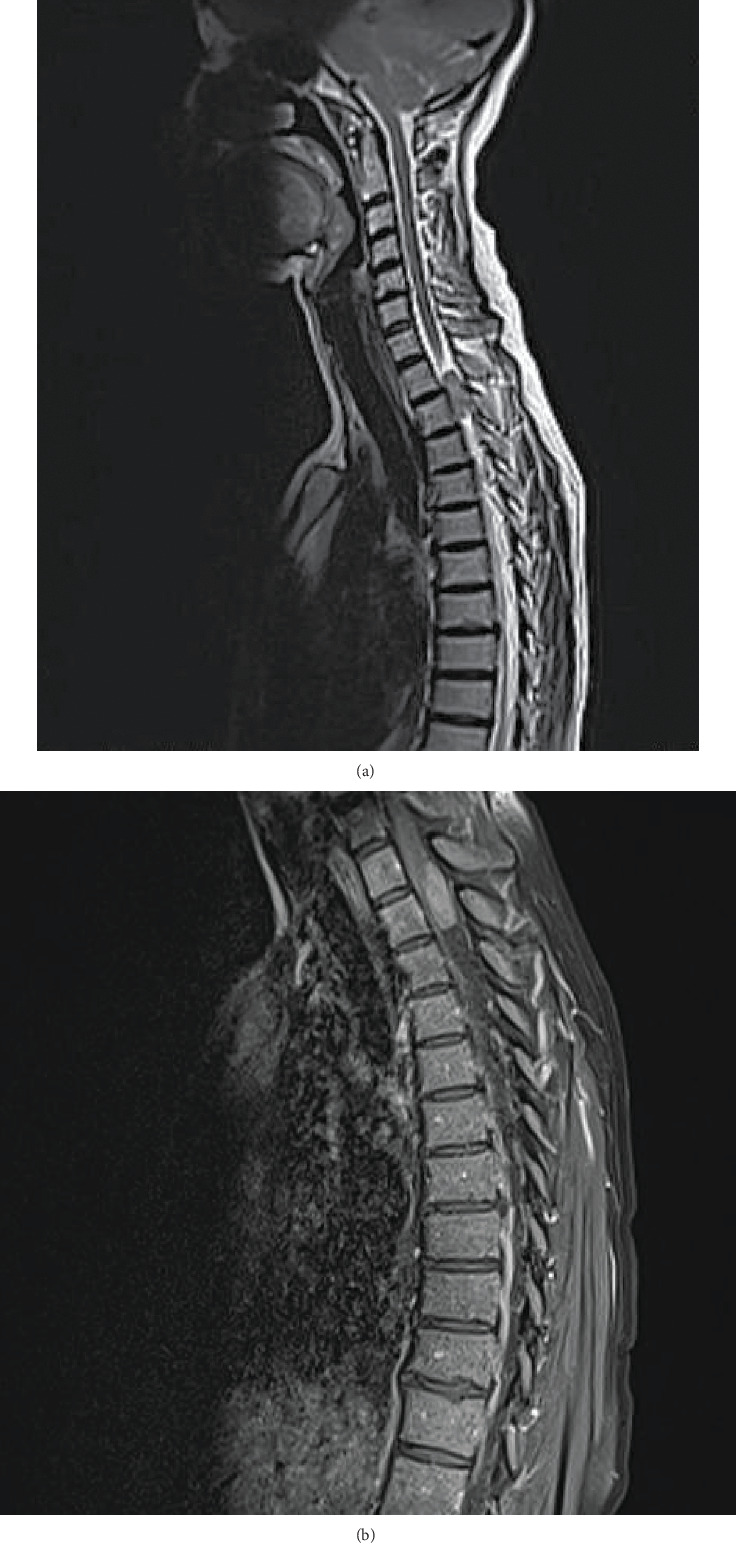
Contrast-enhanced magnetic resonance imaging of the thoracic spine before surgery. (a) Sagittal T_2_-weighted sequence demonstrates a small mass in the upper thoracic spine with primarily high T_2_ signal. (b) Upon contrast application T_1_-weighted sequence demonstrates strong, quite homogeneous mass enhancement.

A 46-year-old woman presented to the Neurosurgery Department of University Medical Center in July 2018 with an impaired sense of pain and temperature in the anterior torso below the sternum. The patient complained of the numbness of both legs progressing from about nine months and the segmental pain in the back radiating to the right arm, induced by pressure and appearing during sports activity—horse riding and running. The patient has been suffering from micturition disorders for several weeks. Physical examination showed the abolition of abdominal reflexes, symmetric cloning of tendon reflexes, and impaired sensation in both legs, with no motor deficits. Laboratory blood tests were requested: they were all normal (RBC 4,5 10^6^/*μ*L; WBC 7,5 10^3^/*μ*L; PLT 263 10^3^/*μ*L; HGB 14 g/dL; HCT 41,1; MCV 91,3%; ALAT 15,0 U/L; ASPAT 26,0 U/L; creatinine 0,76 mg/dL; glucose 87,0; bilirubin 0,8 mg/dL; anti-HCV negative; HbsAg negative). The patient negated any comorbidities and ailments. sagittal and axial T_1_ and T_2_ and gadolinium-enhanced magnetic resonance images (MRI) of the thoracic spine were performed. MRI revealed an oval focal intradural extramedullary lesion significantly compressing the spinal cord (ap 9.4 × si 25 mm), located posterolateral at the Th1-Th2 level. The tumor was isointense on T_1_-weighted image and demonstrated heterogeneously raised hyperintense T_2_ signal. Upon contrast application, the T_1_-weighted sequence demonstrated strong, nearly homogeneous mass enhancement (Figures 1(a) and 1(b)). The patient was hospitalized and operated urgently. The clinical image strongly suggested the meningioma due to its location and MRI characteristics. Meningiomas arise from arachnoid cap cells embedded in dura near the spinal nerve root sleeve. Their predominant spinal canal location is lateral. They are more common in women (up to 85% of cases) and located in the thoracic region of the spine (80%). They present in an oval shape the majority of cases [3]. In 75% of meningiomas, calcifications were registered. [3]. These features led to presurgical planning for meningioma resection. Surgical treatment involved a posterior approach with Th1-Th2 laminectomy and the gross total tumor resection. Pathological examination revealed schwannoma conventional Antoni type A, WHO grade I, with a clearly low proliferation index and positive S-100 reaction. Schwannomas are composed of Schwann cells with fibrous tissue. These tumors may show cystic degeneration and hemorrhage. They usually displace nerve roots [3]. Schwannomas are further classified into two types based on distinct histological patterns—Antoni A (Type 1) and B (Type 2) tumors [4]. When present, heterogeneity of the tumor has been shown to correlate histologically with a greater ratio of Antoni B tissue than Antoni A. On MR imaging, Type 1 predominant tumors tend to be smaller and homogeneous while heterogeneous tumors (with or without cystic degeneration) tend to have higher proportions of Type 2 tissue [4]. In the presented case, the pathological examination confirmed a Type 1 schwannoma.

**Figure 2:**
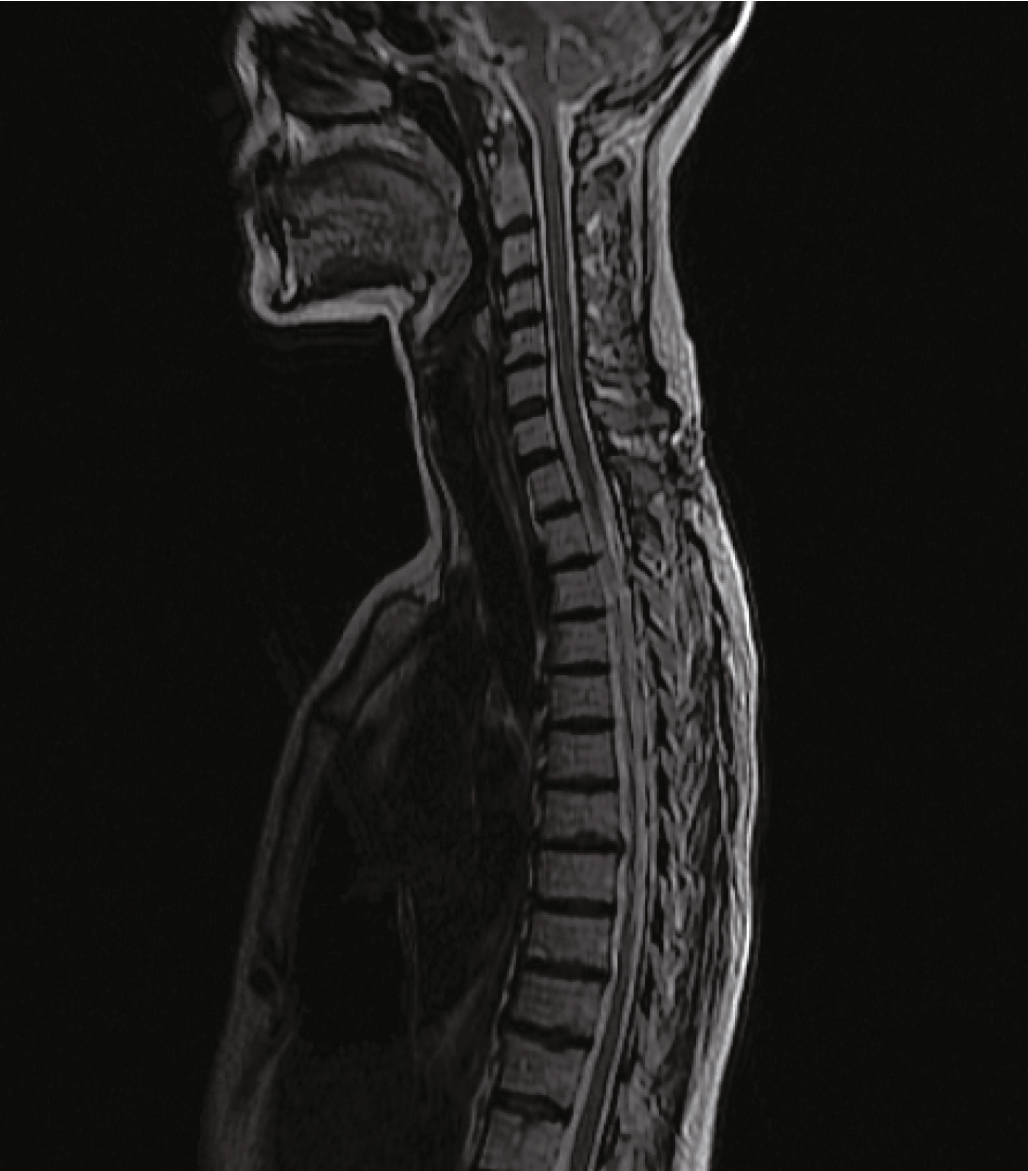
Sagittal T_2_-weighted magnetic resonance imaging of the thoracic spine after surgery.

Radical tumor resection was confirmed on postoperative MRI scans (Figure 2). No tumor recurrence was noted during a follow-up of well over 9 months. During the follow-up period, the patient completely recovered all neurologic deficits after the surgery.

## 3. Discussion

MRI differences which are important in differentiation between intraspinal schwannoma and meningioma are tumor location and shape, mean size, dural contact characteristic signal intensity on T_1_ and T_2_, and enhancement pattern [2]. In the case under discussion, the MRI images and neurologic symptoms suggested the most likely the meningioma. The female sex and age of the patient, the oval shape of the tumor, and its location in the thoracic region were quite typical for meningioma. However, the size of the lesion was not typical for meningioma (mean size for meningiomas are 1.47+/-0.36 cm) [2] and only fragmentary dural base support was visible on MR images. No dumbbell shape and no intervertebral foramen widening were presented. Schwannomas demonstrate typical MRI features of T_1_ iso-to-hypointensity, T_2_ hyperintensity, or fluid signal intensity [4]. In the presented case, fluid signal intensity on T_2_-weighted images and isointensive signal on T1-weighted images might indicate meningioma or schwannoma [2, 5]. Meningiomas homogeneously enhance after contrast administration; however, in schwannomas, enhancement upon contrast application is variable [4]. The most common initial symptom of spinal tumors is pain, followed by a loss of nerve function [6]. In the case under discussion, a very useful piece of information in diagnosis and surgical planning would be the classification of character and timing of the pain reported by the patient. A crucial symptom of schwannoma in this case was the pain induced by pressure at the Th1-Th2 spinal level and appearing and intensifying during sports activity. Approximately 75% of all patients with peripheral nerve sheath tumors have pain in some settings. The distinction of the pain at rest versus other pain characteristics seems to be crucial for the diagnosis: the rest pain is reported only by 5% of patients with schwannomas while the pain induced by pressure is observed in 95% of patients with benign nerve sheath tumors [5]. Pain appearing during physical activity seems to be also an important symptom of spinal schwannoma. Pain is a more frequent symptom in schwannomas than in meningiomas. Schwann cells are involved in dynamic interactions with their associated axons, many of which may promote the development of neuropathic pain in schwannoma [6].

## 4. Conclusion

This case confirms that total tumor resection is associated with better functional outcomes after resection of tumors less than 5 cm in maximum dimension. The role of tumor size has been the object of extensive investigation in the literature, and this factor has been found to be a good prognostic indicator [7]. Histopathological examination of a surgical specimen is the key to confirming differential diagnosis between schwannoma and meningioma. However, there are classical MRI characteristics that can help formulate appropriate differential diagnosis before surgery. While common MRI characteristics for schwannomas and meningiomas exist, diagnosis by imaging alone remains challenging. The pain (its classification of the character and the timing) seems to be an important predictive symptom in the differential diagnosis of spinal schwannoma and meningioma in clinical challenging cases when MRI features are not classical and unequivocal.

## Data Availability

Data are available on request through the author.
